# A simulation-based assessment of the ability to detect thresholds in chronic risk concentration-response functions in the presence of exposure measurement error

**DOI:** 10.1371/journal.pone.0264833

**Published:** 2022-03-11

**Authors:** Garrett Glasgow, Bharat Ramkrishnan, Anne E. Smith

**Affiliations:** 1 NERA Economic Consulting, San Francisco, California, United States of America; 2 NERA Economic Consulting, Washington DC, District of Columbia, United States of America; Indian Institute of Technology Hyderabad, INDIA

## Abstract

An important question when setting appropriate air quality standards for fine particulate matter (PM_2.5_) is whether there exists a “threshold” in the concentration-response (C-R) function, such that PM_2.5_ levels below this threshold are not expected to produce adverse health effects. We hypothesize that measurement error may affect the recognition of a threshold in long-term cohort epidemiological studies. This study conducts what is, to the best of our knowledge, the first simulation of the effects of measurement error on the statistical models commonly employed in long-term cohort studies. We test the degree to which classical-type measurement error, such as differences between the true population-weighted exposure level to a pollutant and the observed measures of that pollutant, affects the ability to statistically detect a C-R threshold. The results demonstrate that measurement error can obscure the existence of a threshold in a cohort study’s C-R function for health risks from chronic exposures. With increased measurement error the ability to statistically detect a C-R threshold decreases, and both the estimated location of the C-R threshold and the estimated hazard ratio associated with PM_2.5_ are attenuated. This result has clear implications for determining appropriate air quality standards for pollutants.

## Introduction

Numerous epidemiological studies over the past several decades have found a statistical association between PM_2.5_ (atmospheric particulate matter with a diameter of 2.5 micrometers or less) and mortality, with the strongest effects usually reported from chronic exposure studies that compare survival outcomes of cohorts in different communities with differing ambient concentrations of PM_2.5_ [1–7]. Moving beyond these studies, determining how the relationship between PM_2.5_ and mortality might change as exposure changes is a fundamental challenge important to determining whether and to what extent tightening air quality standards for PM_2.5_ will result in public health benefits. Such determinations are complicated by the presence of potential exposure measurement error, as has been suggested by other authors [8,9].

The major cohort epidemiological analyses done to date have involved people who were enrolled at the beginning of a study, with data such as health markers and demographic information gathered at enrollment. The cohort directors followed the study subjects over time and marked when certain health events occurred, such as a medical diagnosis or death. Study authors also estimated the subjects’ air pollution exposure and conducted statistical analyses (often Cox proportional hazards modeling) to determine whether there was an association between the pollutant exposure and the health effect in the specific cohort of people. The exposure estimation is rarely at the personal level; exposure is often assigned at a community level, meaning that all members of one community (such as a census tract, a zip code, or a whole city) are assigned the same estimate of pollution exposure. Recently air quality modeling methods have been used to develop much more refined locational exposure estimates for epidemiological studies, but also without personal exposure data.

Setting aside thorny questions of determining whether the relationship between PM_2.5_ and mortality is causal, the “shape” of the concentration-response (C-R) function underlying that association is an important consideration when setting an adequately-protective ambient air quality standard for PM_2.5_. A C-R relationship’s shape can determine whether changes in PM_2.5_ at relatively low concentrations will have the same, greater, or lesser effects on health risk as changes at concentrations that contributed to an observed association. Although shape can have many forms, a common shape of interest to policy makers is whether there may be an effect “threshold” such that PM_2.5_ levels below this threshold are not expected to produce adverse health effects.

A majority of the previous research on the relationship between long-term PM_2.5_ exposure and mortality in U.S.-based cohorts has not detected such a threshold. There are at least three possible explanations for these findings. The first is that there is in fact no threshold in the C-R function, and any concentration of PM_2.5_ will produce an adverse health effect. A second possible explanation is that a C-R threshold does exist, but the historical PM_2.5_ concentrations in these studies have almost always been above the C-R threshold. For example, while the U.S. population-weighted annual average PM_2.5_ concentration in 2016 was 9.0 μg/m^3^ [10], less than 10 percent of the PM_2.5_ concentrations measured from 1999–2000 in the American Cancer Society (ACS) study fell below this level [3]. A C-R threshold near the bottom of the range measured by these studies might be difficult to detect, even with quite reliable exposure estimates. A third possibility, and the subject of this paper, is that a C-R threshold does exist even within the range of PM_2.5_ concentrations observed in the study, but it is obscured by measurement error in the estimated PM_2.5_ concentrations.

There are two major types of measurement error that could affect the PM_2.5_ exposure estimate: Berkson-type error and classical-type error [11]. Berkson-type error typically arises from differences between each individual’s personal PM_2.5_ exposure and the community-average personal exposure as measured by a PM_2.5_ monitor or estimated by a model. With Berkson-type error the true PM_2.5_ exposure for each individual is randomly distributed around the measured PM_2.5_ value, but the true population-weighted mean PM_2.5_ value is accurately measured and will thus not lead to bias in estimates of the C-R function, although it is expected to increase the variance of the estimate. Classical-type error will arise if there are differences between the true population-weighted exposure level and the observed measures of PM_2.5_. It is well known that classical-type measurement error can lead to biased estimates of a C-R function’s parameters [e.g., 12–18]. In summarizing previous research on this topic, Rhomberg et al. [9] conclude “[t]he majority of the literature, both theoretical and experimentally based, indicates that measurement error results in the masking of a threshold, and that even when exposure measurement error is not large enough to completely linearize a truly threshold exposure-response relationship, bias still exists.” This study adds to that literature by conducting what is, to the best of our knowledge, the first simulation of the effects of measurement error on the statistical models commonly employed in long-term cohort studies.

One common approach to testing for thresholds in C-R functions is to estimate a statistical model relating a pollutant to mortality, assuming the pollutant has no effect on mortality below a specified threshold, and a linear effect above the threshold such that increasing levels of the pollutant increase the risk of mortality. The fit of the threshold model is then tested against the fit of a no-threshold model [e.g., 19,20]. The statistical model used to test for a threshold in this paper is the Cox proportional hazards model, a commonly-employed model in the study of long-term cohort studies that assesses the relationship between a risk factor (such as air pollution) and survival time. Another approach to testing for thresholds in C-R functions is to estimate a non-parametric (spline) regression of the relative risk of mortality on the exposure range of the pollutant, and examine the resulting plot for evidence of a threshold [e.g., 21]. We examine the effect of measurement error on both approaches.

We note that simulation is an important supplement to standard epidemiological investigations that rely on observed rather than simulated evidence. In standard epidemiology studies with observed cohort data the true mean PM_2.5_ value for each community and the true shape of the C-R relationships are unknown to epidemiological researchers, so it is not possible to determine the effect of classical measurement error on the ability to detect a C-R threshold. In this study we test the degree to which measurement error affects the ability to statistically detect a C-R threshold under a variety of different conditions (different C-R thresholds, levels of measurement error, and hazard ratios), using a large, simulated cohort. We also test the degree to which estimates of the level of a true C-R threshold and of the true hazard ratio associated with PM_2.5_ are affected by differing amounts of classical measurement error.

## Methods

**Fig 1:**
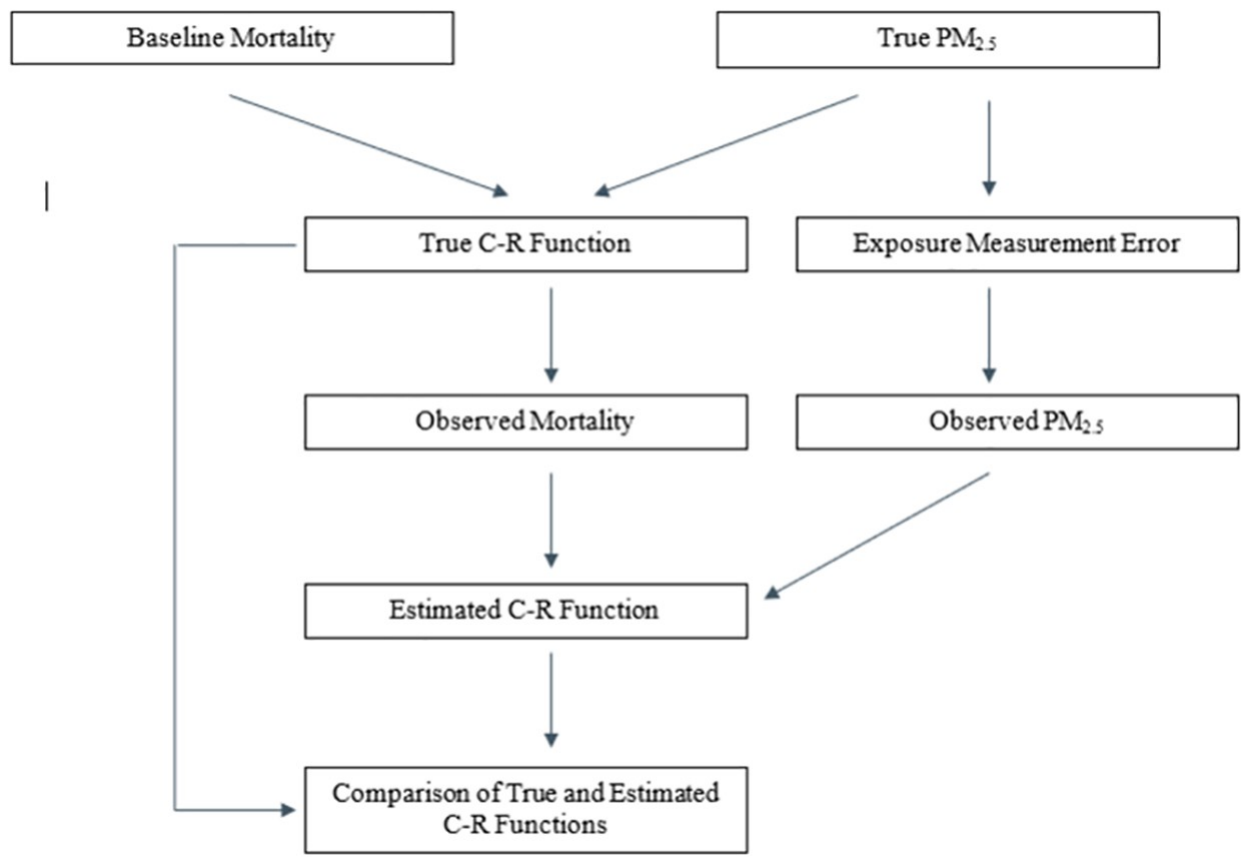
Flow chart of simulation structure.

Our simulations are described in detail below. A flow chart representing the structure of our simulations is presented in Fig 1.

### Generating the simulated cohorts

We generated a cohort of simulated individuals that was tracked for 20 years, from 2000 to 2020. Our simulated cohort consists of populations from 100 different hypothetical cities, each with 20,000 simulated individuals, for a total cohort size of 2 million individuals.

The baseline mortality rate for the simulated individuals in our cohort was calculated from cohort life tables compiled by the US Social Security Administration [22]. These life tables give the probability of mortality at each age based on birth year and sex, with birth year reported in 10-year increments from 1900 to 2100. We used linear interpolation to assign mortality probabilities for birth years that fell between those in the life table.

For each simulated individual, a birth year was calculated by subtracting the age assigned to that individual from the first calendar year of the simulation (2000), and the probability of mortality for each simulated individual in each year of the simulation was assigned based on the mortality probabilities calculated above.

For each simulated individual in each year of the simulation, mortality was determined by a random draw from a uniform distribution in the range of 0 to 1. If the random draw was less than the probability of mortality in that year for a simulated individual, that individual was recorded as dying in that year.

To clarify the influence of measurement error on the detection of a threshold in a C-R function, we limited the cohort we generated to only males aged 60 at the start of the simulation. We did this to eliminate variation in the baseline mortality rate based on age and sex that would otherwise make the detection of a threshold more difficult. Variation in age and sex could also be introduced into the cohort and is an area for future study.

### PM_2.5_ levels and the C-R functions

To create a dataset with PM_2.5_ conditions for 100 hypothetical cities that would be generally consistent with those in the U.S., we used the weighted annual mean of daily PM_2.5_ concentrations from all 229 core-based statistical areas (CBSAs) in the EPA’s AQS database from 2000 to 2016, with the weighted annual mean calculated as the average of the quarterly averages of the 24-hour values [23]. We used linear extrapolation to extend the data for each CBSA out to 2020 to create a twenty-year data set. We organized the data into quartiles, and randomly selected 25 sets of PM_2.5_ values from each quartile, which were then randomly assigned to the 100 hypothetical cities. For this analysis, we have assumed no time-variation in PM_2.5_ concentrations and have used the simple average PM_2.5_ concentration from the 2000–2020 period of actual monitored values, plus a random draw from a uniform distribution bounded between -1 and 1 μg/m^3^ to arrive at the PM_2.5_ concentrations for the hypothetical cities. These PM_2.5_ values were then used in our simulations as the “true” population-weighted average exposure. The additional effect of time-varying PM_2.5_ on simulation results is an area for future study.

In each hypothetical city in each year, true PM_2.5_ concentration was assumed to influence the probability of mortality. Specifically, the probability of mortality (*P*_*ijt*_) for simulated individual *i* in city *j* at time *t* was calculated as:

$$P_{ijt}=B_{ijt}\;\times h^{{PM}_{jt}}$$ (1)

where *B*_*ijt*_ is the baseline probability of mortality for simulated individual *i* in city *j* at time *t*, *PM*_*jt*_ is the PM_2.5_ level in city *j* at time *t*, and *h* is the hazard ratio (HR) related to exposure to PM_2.5_. This equation defines a linear C-R function on the log hazard scale. Further, the entire risk from PM_2.5_ exposure in this C-R function occurs in the same year as exposure (i.e., there are no lags or cumulative effects to complicate the detection of the true C-R shape).

In our simulations we test C-R functions with well-defined (i.e., “hockey-stick”), population-wide thresholds, such that PM_2.5_ below the threshold has no effect on mortality, and PM_2.5_ above the threshold has a linear effect. To do this we subtract the threshold from the PM_2.5_ level in each city and year, setting “effective” PM_2.5_ to zero if this calculation results in a negative number. This modified measure of PM_2.5_ is then substituted in for *PM*_*jt*_ in the equation above. This produces a true C-R function that is zero below the threshold, and linear above the threshold.

We ran simulations for a wide range of alternative C-Rs. We examined three alternative “true” PM_2.5_ threshold levels: 7 μg/m^3^, 8.5 μg/m^3^, and 9.5 μg/m^3^. These thresholds correspond approximately to the 15^th^, 40^th^, and 55^th^ percentiles of our assumed true PM_2.5_ exposure levels and were selected to test various threshold levels approximately at or below the mean level of PM_2.5_. In our simulations we considered all combinations of these three C-R thresholds with five alternative “true” HRs above the threshold (1.0025, 1.005, 1.01, 1.02, and 1.05 per μg/m^3^), for a total of 15 different C-Rs.

### Measurement error

The measurement error we examine in our simulations allows the “observed” average PM_2.5_ exposure assigned to a hypothetical city to deviate from the “true” average PM_2.5_ exposure experienced by the individuals in these cities. This type of measurement error could arise if, for example, the monitoring location used to measure PM_2.5_ levels does not reflect the “true” population-weighted average PM_2.5_ levels experienced by the population. This is a type of classical measurement error (as opposed to Berkson measurement error, under which individual exposures vary randomly around the population average exposure).

The “observed” PM_2.5_ measures for each hypothetical city were created by adding a random draw to the city’s “true” PM_2.5_ level. The random draws came from a truncated normal distribution with bounds at ± 4 μg/m^3^. Increasing amounts of measurement error were created by increasing the standard error on this truncated normal distribution. We considered draws of measurement error from distributions with standard deviations of 1, 2, and 4 μg/m^3^.

For each standard deviation, we drew 100 different sets of “observed” PM_2.5_ values for each of the hypothetical cities in our simulations. We then ran 100 versions of the epidemiological models described below for each simulated cohort, once for each of the 100 different sets of “observed” PM_2.5_ values. The same sets of “observed PM_2.5_” were used for each set of epidemiological models tested below. Thus, any variation in the ability to detect a C-R threshold across different “true” C-R functions is not due to variation in the measurement errors used from test to test.

### Empirical tests

In our simulations we examined all combinations of true C-Rs and measurement error. There were three “true” PM_2.5_ thresholds (7, 8.5, and 9.5 μg/m^3^), five “true” HRs (1.0025, 1.005, 1.01, 1.02, and 1.05 per μg/m^3^), and three levels of measurement error (distributed as a truncated normal with standard deviations of 1, 2, and 4 μg/m^3^), for a total of 45 combinations of variables. Each of these combinations was represented by a separate simulated set of cohort survival outcome data, on which we ran epidemiological models to estimate the underlying true C-R relationship. For each of these 45 simulated cohort survival outcome datasets, we ran 100 models, one for each of the 100 different sets of “observed” PM_2.5_ values described above.

As a preliminary examination we first fit splines to the simulated data. The dependent variable in our splines was relative risk as calculated from the “observed” mortality in each city, with relative risk defined as 1 for the lowest “observed” level of PM_2.5_. For each of the 45 combinations of threshold, HR, and measurement error, natural cubic splines with four degrees of freedom were estimated on the relative risk and examined for evidence that thresholds in a C-R function would be more difficult to detect as measurement error increases.

Next, for each of these combinations, we ran a series of Cox proportional hazard (PH) models to test for our ability to detect a C-R threshold in the face of measurement error. For each “true” threshold, we created a new PM_2.5_ measure by subtracting the “true” threshold from “observed” PM_2.5_, as described above. This measure of PM_2.5_ was then used to estimate a Cox PH model. For each combination of variables, we also estimated a model that assumed there was no C-R threshold.

We then compared the statistical fits of these models. For each combination of “true” threshold, HR, and level of measurement error, both a model that assumed the true threshold and a no-threshold model were estimated for each of the 100 values of “observed” PM_2.5_. The threshold model and the no-threshold model are not nested, so statistical tests such as the likelihood-ratio test cannot be applied. Thus, we followed the suggestion of Jerrett et al. [20,24] and calculated a test statistic of 2 times the difference in the log-likelihoods between the threshold model and the no-threshold model *(2 × (LL(threshold)–LL(no threshold))*, which was then compared to the likelihood-penalty function applied by the Akaike information criteria (AIC) and Bayesian information criteria (BIC) for one additional parameter, which for the AIC is 2 and for the BIC is ln(n), with n defined as either the number of deaths observed in the data or the number of individuals in the data. A test statistic greater than any one of these values could lead one to conclude that the threshold model is a better fit than the no-threshold model. Some of these tests are more stringent than others (i.e., are less likely to reject the non-threshold hypothesis). The simulation results presented below use the most stringent test standard proposed by Jerrett et al. [24] and compare the difference in the log-likelihoods to the natural log of the number of individuals in the data. Simulation results based on the other test standards proposed by Jerrett et al. [24] are presented in the Supporting Information (SI).

We also examine the estimated C-R threshold levels (based on the best fitting model) for each combination of “true” threshold, HR, and measurement error for each of the 100 values of “observed” PM_2.5_. For each simulated dataset a grid search approach was used to test the fit of various candidate thresholds. We examined alternative threshold estimates in the range ± 4 μg/m^3^ around the “true” PM_2.5_ threshold value, incremented by 1 μg/m^3^. For example, if the “true” threshold was 8.5 μg/m^3^, we examined models that assumed thresholds ranging between 4.5 and 12.5 μg/m^3^, incremented by 1 μg/m^3^. For each potential threshold, we created a new PM_2.5_ measure by subtracting the potential threshold from the “observed” PM_2.5_. This measure of PM_2.5_ was then used to estimate a Cox PH model. The threshold from the model with the best log-likelihood was then reported as the best-fitting threshold.

We also examined the estimated HRs for each combination of “true” threshold, HR, and measurement error.

## Results

### Threshold detection with splines

Examination of splines estimated on each combination of threshold, HR, and measurement error indicates that as measurement error increases, the existence of a threshold becomes more difficult to determine, the estimated threshold location decreases, and the HR becomes attenuated.

**Fig 2:**
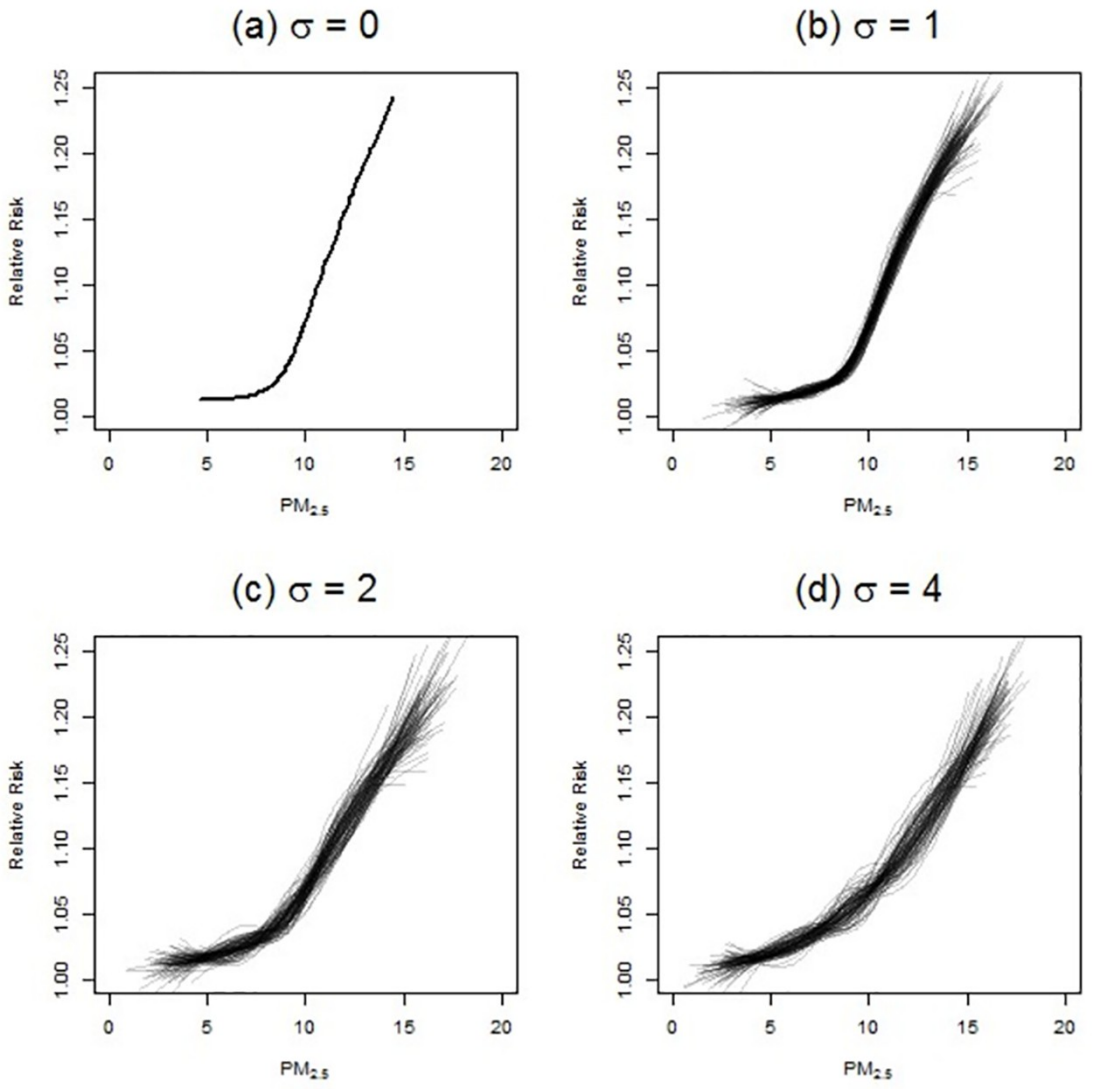
Spline estimates of same “true” C-R function under varying amounts measurement error.

Here we discuss splines estimated on the data produced by simulations with a threshold of 8.5 μg/m^3^ and an HR of 1.05. Fig 2 presents four sets of splines estimates (each estimated with 4 degrees of freedom). One scenario assumes no measurement error, while the other three examine the three levels of measurement error (*σ* = 1, 2, or 4 μg/m^3^). Each panel in Fig 2 presents 100 splines estimated using one of 100 separately and randomly generated sets of city-specific “observed” values of PM_2.5_ for a particular level of measurement error: *σ* = 0, 1, 2, or 4 μg/m^3^. In all 100 iterations, the true C-R function and the true city-specific PM_2.5_ values were the same, with the identical dataset on cohort survival outcomes being used for each spline; only the draws of the measurement error differ across the 100 estimated splines and cause the differences from spline to spline in each panel.

The presence of a threshold in the C-R function at a PM_2.5_ concentration of 8.5 μg/m^3^ is clearly apparent in panel (a) of Fig 2, when there is no measurement error. As measurement error increases, both the apparent level of the threshold and strength of the relationship between PM_2.5_ and mortality are attenuated towards zero. Although some degree of “sublinear” shape is evident when viewing all 100 splines overlaid in a single panel, many of the individual splines have no threshold-like shape at all; some even take the opposite, “supralinear” shape over parts of the C-R function. However, it is misleading to rely on a comparison across 100 draws of measurement error to understand the “model uncertainty” created by measurement error: any actual epidemiological study faces the equivalent of a single draw, with a single resulting C-R estimate. The potential degree of deviation of the shape of that C-R estimate from the true underlying shape can be much greater than the aggregated patterns in Fig 2.

All else being equal, such model uncertainty increases with increasing measurement error, but it can be exacerbated or moderated under different locations of the true threshold (relative to the observed range of concentrations) and different levels of the true HR. Figures equivalent to Fig 2 for every combination of threshold and HR are presented in the SI (S1 to S15 Figs). We note that in some of these cases the evidence of a nonlinear shape dissipates across the 100 measurement error draws (e.g., S3 Fig). We also note that in certain cases, noise in the cohort simulation itself (due to the mortality outcomes being determined by random draws) is sometimes large enough that the splines estimated on the cohort outcomes are unable to detect the presence of a threshold in the true C-R function even when such a threshold exists (e.g., S11 Fig).

Measurement error affects the ability of splines to detect the true shape of the underlying C-R relationship by shifting each observed value of PM_2.5_ horizontally in either a positive or negative direction, thus “flattening out” the relationship between PM_2.5_ and mortality, resulting in non-zero estimates of risk below the C-R threshold. This phenomenon also affects the ability of parametric statistical methods used in cohort studies to produce reliable estimates of the true C-R shape, as we show below.

### Parametric statistical tests for thresholds

Table 1 presents the results from testing the fit of Cox PH models that assume the correct threshold compared to the fit of a no-threshold model for each level of measurement error (*σ* = 1, 2, or 4 μg/m^3^) for the five different HRs considered in the simulations. Results for each of the three C-R thresholds we consider are presented in separate columns. The values in each row indicate the number of times (out of 100 sets of city-specific measurement error assignments) the Cox PH model that assumed the true threshold fit the data better than the no-threshold Cox PH model. The test statistic used is based on 2 times the difference in the log-likelihoods between the threshold and no-threshold models being larger than the natural log of the number of individuals in the data (*2 × ΔLL > ln(n)*).

**Table 1:** Rejection of the no C-R threshold model under varying amounts of measurement error.

	Threshold = 7	Threshold = 8.5	Threshold = 9.5
HR = 1.0025			
σ = 1	0	0	0
σ = 2	0	0	0
σ = 4	0	0	0
HR = 1.005			
σ = 1	13	0	0
σ = 2	4	2	0
σ = 4	5	0	0
HR = 1.01			
σ = 1	1	99	67
σ = 2	3	71	27
σ = 4	3	37	18
HR = 1.02			
σ = 1	96	100	100
σ = 2	67	100	98
σ = 4	43	82	91
HR = 1.05			
σ = 1	100	100	100
σ = 2	94	100	100
σ = 4	79	100	100

Three patterns are apparent in Table 1. First, the ability to detect a threshold increases as the HR increases. Higher hazard ratios lead to a more rapid increase in risk once the C-R threshold is crossed, making it easier to distinguish the boundary between zero and positive risk. For HRs less than 1.01 there was little detection of the true threshold at all, even for the highest threshold level tested.

Second, the ability to detect a threshold tends to increase when the threshold is located higher in the range of true PM_2.5_ exposures experienced by the cohort. Recall that the thresholds of 7, 8.5, and 9.5 μg/m^3^ correspond approximately to the 15^th^, 40^th^, and 55^th^ percentiles of the true PM_2.5_ exposure levels, respectively. Our simulations find that for HRs of 1.01 or higher (the HR levels for which a meaningful fraction of the simulations does detect the threshold), as the true threshold increases, the Cox PH model that assumes a threshold is more likely to detect it, especially when the threshold is increased from 7 to 8.5 μg/m^3^. This pattern does not seem to hold for the lower HRs.

Third, for the higher HR and threshold levels, the ability to detect a threshold decreases as measurement error increases (as σ grows larger). In all cases, the number of times the threshold model fit the data better than the no-threshold model decreased as the amount of measurement error increased.

Simulation results based on the other test standards proposed by Jerrett et al. [24] are presented in the SI (S1 and S2 Tables). These alternative results support the conclusions we describe here.

### Estimated threshold levels

Tables 2–4 present the results from estimating the location of the C-R threshold based on the best-fitting Cox PH model across a range of candidate thresholds and across the 100 different sets of “observed” values of PM_2.5_. For the three “true” C-R threshold levels that we analyzed (each in a separate table) we present results for each combination of HR and level of measurement error. The values in each row indicate the number of times out of 100 each potential threshold (as identified by the column headers) was found to produce the best-fitting Cox PH model. As there were 100 separate sets of observed PM_2.5_ modeled for each row, the numerical values in each row sum to 100.

**Table 2:** Best-fitting C-R threshold level under varying amounts of measurement error, true threshold = 7.

	Potential C-R Threshold Tested for Goodness of Fit							
	3	4	5	6	7	8	9	10
HR = 1.0025								
σ = 1	1		4	7	20	26	33	9
σ = 2	7	3	5	14	16	22	11	14
σ = 4	11	9	17	11	9	14	9	11
HR = 1.005								
σ = 1				1	23	63	13	
σ = 2	1	2	5	18	24	31	18	1
σ = 4	6	6	6	12	24	19	15	7
HR = 1.01								
σ = 1	2	2	10	50	35	1		
σ = 2	20	18	28	17	11	6		
σ = 4	28	20	17	8	13	8	5	
HR = 1.02								
σ = 1			1	11	83	5		
σ = 2		4	17	36	32	8	3	
σ = 4	9	17	22	16	19	14	3	
HR = 1.05								
σ = 1				24	75	1		
σ = 2			26	49	19	5	1	
σ = 4	6	29	25	13	17	6	4	

**Table 3:** Best-fitting C-R threshold level under varying amounts of measurement error, true threshold = 8.5.

	Potential C-R Threshold Tested for Goodness of Fit							
	4.5	5.5	6.5	7.5	8.5	9.5	10.5	11.5
HR = 1.0025								
σ = 1		3		3	24	48	20	2
σ = 2	4	5	5	12	15	29	15	13
σ = 4	5	4	7	8	16	12	19	10
HR = 1.005								
σ = 1	3	1	2	25	53	15	1	
σ = 2	5	10	18	19	25	13	7	2
σ = 4	19	6	11	19	20	5	10	4
HR = 1.01								
σ = 1				1	63	34	2	
σ = 2		1	3	25	53	15	3	
σ = 4	6	7	14	25	27	13	3	5
HR = 1.02								
σ = 1				23	77			
σ = 2			14	56	26	4		
σ = 4	3	11	31	29	16	10		
HR = 1.05								
σ = 1				21	79			
σ = 2			7	67	22	3	1	
σ = 4		6	29	40	21	3		1

**Table 4:** Best-fitting C-R threshold level under varying amounts of measurement error, true threshold = 9.5.

	Potential C-R Threshold Tested for Goodness of Fit							
	5.5	6.5	7.5	8.5	9.5	10.5	11.5	12.5
HR = 1.0025								
σ = 1	56	11	12	14	5		1	1
σ = 2	39	11	13	23	9	2	2	1
σ = 4	41	12	13	9	7	8	5	3
HR = 1.005								
σ = 1	2	8	44	40	6			
σ = 2	26	16	18	23	14	2	1	
σ = 4	30	12	11	22	5	11	5	4
HR = 1.01								
σ = 1			5	50	43	2		
σ = 2	8	6	29	40	12	4	1	
σ = 4	12	12	26	24	19	3	3	1
HR = 1.02								
σ = 1				17	81	2		
σ = 2		1	15	50	25	9		
σ = 4		10	23	34	15	8	10	
HR = 1.05								
σ = 1				11	88	1		
σ = 2			8	62	25	5		
σ = 4		3	20	48	12	10	6	1

It is apparent in examining Tables 2–4 that measurement error makes detection of the true C-R threshold difficult. The effect of measurement error on the ability to detect a threshold is most clear when considering the higher HRs in the simulation (HR = 1.02 or 1.05). There the best-fitting Cox PH model indicates the “true” threshold in a majority of the 100 tests for the lowest level of measurement error (*σ* = 1), and underestimates of the threshold become more common as measurement error increases. For lower HRs the patterns become less clear, and for very low HRs overestimates of the threshold are frequent. However, for most combinations of HR, threshold, and levels of measurement error we examine, underestimates of the threshold are more common than overestimates.

### Estimated hazard ratios

**Fig 3:**
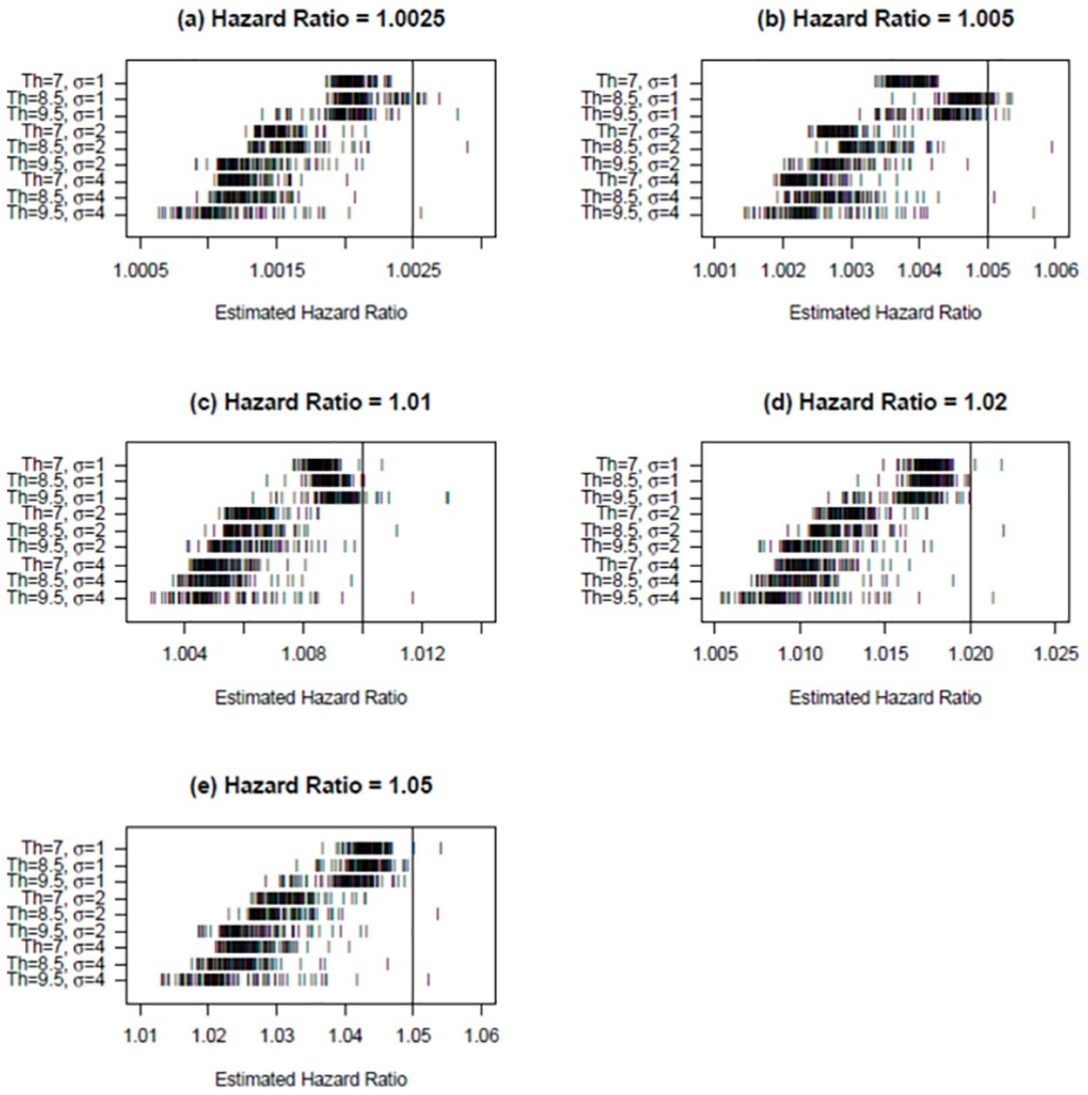
Attenuation in hazard ratios under varying amounts of measurement error and different “true” C-R thresholds.

Fig 3 considers the HRs estimated by the best-fitting Cox PH model across the 100 different “observed” values of PM_2.5_. Each panel of Fig 3 presents the results for one hazard ratio, which is indicated by the solid vertical line on the panel. For each combination of “true” threshold and level of measurement error (identified on the vertical axis of the panel), each tick mark in a panel indicates an estimated hazard ratio.

Two patterns are apparent when examining Fig 3. First, as expected, the estimated HRs become more attenuated as the level of measurement error increases. This pattern holds true regardless of which HR or threshold is considered. Second, the amount of attenuation related to measurement error tends to be higher as the threshold increases, especially at higher levels of measurement error.

In most cases, the 95 percent confidence interval on the HRs presented in Fig 3 would reject the “true” HR as too high. These results are presented in Table 5. Each numerical result indicates the number of times the upper limit of the 95 percent confidence interval on the HR in the best-fitting Cox PH model falls below (fails to cover) the “true” HR assumed in that simulation. Table 5 shows that the best-fitting Cox PH model is more likely to reject the “true” HR as too high as measurement error increases across all three “true” thresholds, especially for larger values of the “true” HR.

**Table 5:** True hazard ratio above upper limit of estimated hazard ratio, under varying amounts of measurement error and different “true” C-R thresholds.

	Threshold = 7	Threshold = 8.5	Threshold = 9.5
HR = 1.0025			
σ = 1	0	0	0
σ = 2	86	51	58
σ = 4	95	91	100
HR = 1.005			
σ = 1	72	2	20
σ = 2	99	91	94
σ = 4	100	96	90
HR = 1.01			
σ = 1	85	42	19
σ = 2	100	99	95
σ = 4	100	99	95
HR = 1.02			
σ = 1	98	90	95
σ = 2	100	100	100
σ = 4	100	99	99
HR = 1.05			
σ = 1	99	98	99
σ = 2	100	100	100
σ = 4	100	100	99

## Discussion

In this study we generated simulated cohorts with C-R functions for PM_2.5_ that varied both in the location of a C-R threshold and the HR associated with PM_2.5_. We found that as measurement error increased, our ability to statistically detect a threshold decreased, and both the estimated location of the threshold and the estimated HR were attenuated. Beyond these general observations, examination of nonlinear splines revealed that measurement error could lead to a variety of different estimated shapes for the C-R function for the same underlying “true” C-R function. Since the true level of measurement error is unknown, this introduces considerable model uncertainty into the estimated shapes of C-R functions from epidemiological studies using observed data.

The results of our study are consistent with other simulation-based studies that have assessed how measurement error affects estimates of the shape of a pollutant’s C-R relationship. Some studies have demonstrated that measurement error leads to attenuated estimates of the slope of a C-R function, without reference to the shape of the C-R function [12,13]. Other simulation-based studies have also examined the shape of the C-R function and have demonstrated that exposure measurement error can “linearize” and “flatten” estimates of a segmented linear, or “hockey stick” C-R function such as the ones we examine here [8,9,14–18]. As in our study, these studies find that exposure measurement error leads to (1) attenuated estimates of the slope of a C-R function, (2) estimates that suggest a C-R function is linear when it in fact has a threshold, and (3) underestimates of the location of a threshold in a C-R function. To the best of our knowledge, our study is the first simulation-based study to test for the magnitude of these effects using the methods commonly employed in cohort studies of the relationship between long-term PM_2.5_ exposure and mortality. We do this by simulating prospective cohort survival data and applying Cox proportional hazard and spline regression methods to assess C-R shape. As our study is focused on the methods commonly employed in cohort studies, it does not consider statistical approaches that might better address concerns with measurement error, such as Bayesian methods that incorporate knowledge or beliefs about the measurement error process [25].

Our study does not consider other sources of uncertainty and variability that might also affect the ability to detect a threshold in a C-R function, such as Berkson measurement error, confounding variables, differences in the properties of PM_2.5_ in different locations, or the effects of pollutants other than PM_2.5_. For instance, Moolgavkar et al. [26] simulated a prospective cohort and applied a Cox PH model to study the potential for spurious small associations to be detected (or masked) if the largest risk factors (e.g., smoking) violate the proportional hazards assumption, and found that inadequate control for strong, time-dependent confounding produces unreliable estimates of risk. Our simulations show that even with a single risk factor and very “clean” data that meets the proportional hazards assumption, measurement error alone can make estimates of the shape of the C-R function unreliable. The difficulties we note in our paper would probably be greatly exacerbated if we were to also consider confounding covariates, or the other sources of uncertainty and variability listed above) [26]. The simulations we describe here could be extended to cover these and other issues.

Most previous epidemiological research on the relationship between PM_2.5_ and mortality that has considered the possibility of a C-R threshold has not detected a statistically significant threshold, and risk analyses based on these epidemiological results then often assume that no such threshold exists. Our results show that the inability of previous research to detect a C-R threshold may be due to measurement error, rather than the nonexistence of such a threshold. Put another way, “because of the prevalence of exposure measurement error in epidemiology data and lack of reliable error-mitigating techniques, conclusions about the linearity of the exposure-response curve must be examined carefully and treated with some scepticism” [9]. This has obvious implications for determining appropriate air quality standards, since most policy makers have relied on risk analyses that have assumed no C-R thresholds exist.

## Conclusions

To the best of our knowledge, this is the first simulation-based study to examine the effect of classical-type measurement error in pollutant exposure on estimates of the shape of a pollutant’s C-R function using simulations of prospective cohort data, and then applying the statistical models commonly employed in long-term cohort studies of the relationship between long-term PM_2.5_ exposure and mortality. The results of our study demonstrate that exposure measurement error obscures the existence of a threshold in the C-R function when such a threshold in fact exists and leads to attenuated estimates of both the estimated location of the C-R threshold and the estimated hazard ratio associated with PM_2.5_. These results have clear implications for determining appropriate air quality standards for pollutants. The extent of measurement error in estimates of pollutant exposure should be more carefully quantified, and its potential effects on uncertainty in the shape of the C-R functions merits consideration by policy makers when setting air quality standards.

## Supporting information

S1 Fig Spline estimates of same “true” C-R function under varying amounts of measurement error (threshold 7 μg/m^3^, hazard ratio 1.0025). (TIF)

S2 Fig Spline estimates of same “true” C-R function under varying amounts of measurement error (threshold 7 μg/m^3^, hazard ratio 1.005). (TIF)

S3 Fig Spline estimates of same “true” C-R function under varying amounts of measurement error (threshold 7 μg/m^3^, hazard ratio 1.01). (TIF)

S4 Fig Spline estimates of same “true” C-R function under varying amounts of measurement error (threshold 7 μg/m^3^, hazard ratio 1.02). (TIF)

S5 Fig Spline estimates of same “true” C-R function under varying amounts of measurement error (threshold 7 μg/m^3^, hazard ratio 1.05). (TIF)

S6 Fig Spline estimates of same “true” C-R function under varying amounts of measurement error (threshold 8.5 μg/m^3^, hazard ratio 1.0025). (TIF)

S7 Fig Spline estimates of same “true” C-R function under varying amounts of measurement error (threshold 8.5 μg/m^3^, hazard ratio 1.005). (TIF)

S8 Fig Spline estimates of same “true” C-R function under varying amounts of measurement error (threshold 8.5 μg/m^3^, hazard ratio 1.01). (TIF)

S9 Fig Spline estimates of same “true” C-R function under varying amounts of measurement error (threshold 8.5 μg/m^3^, hazard ratio 1.02). (TIF)

S10 Fig Spline estimates of same “true” C-R function under varying amounts of measurement error threshold 8.5 μg/m^3^, hazard ratio 1.05). (TIF)

S11 Fig Spline estimates of same “true” C-R function under varying amounts of measurement error (threshold 9.5 μg/m^3^, hazard ratio 1.0025). (TIF)

S12 Fig Spline Estimates of same “true” C-R function under varying amounts of measurement error (threshold 9.5 μg/m^3^, hazard ratio 1.005). (TIF)

S13 Fig Spline estimates of same “true” C-R function under varying amounts of measurement error (threshold 9.5 μg/m^3^, hazard ratio 1.01). (TIF)

S14 Fig Spline estimates of same “true” C-R function under varying amounts of measurement error (threshold 9.5 μg/m^3^, hazard ratio 1.02). (TIF)

S15 Fig Spline estimates of same “true” C-R function under varying amounts of measurement error (threshold 9.5 μg/m^3^, hazard ratio 1.05). (TIF)

S1 Table Rejection of the no C-R threshold model under varying amounts of measurement error (2 × ΔLL > 2). (PDF)

S2 Table Rejection of the no C-R threshold model under varying amounts of measurement error (2 × ΔLL > ln(nevents)). (PDF)
