# Supplementary figures and images for: A simulation-based assessment of the ability to detect thresholds in chronic risk concentration-response functions in the presence of exposure measurement error
Source: PLoS One. 2022 Mar 11;17(3):e0264833. doi: 10.1371/journal.pone.0264833 (PMC8916630; doi:10.1371/journal.pone.0264833)

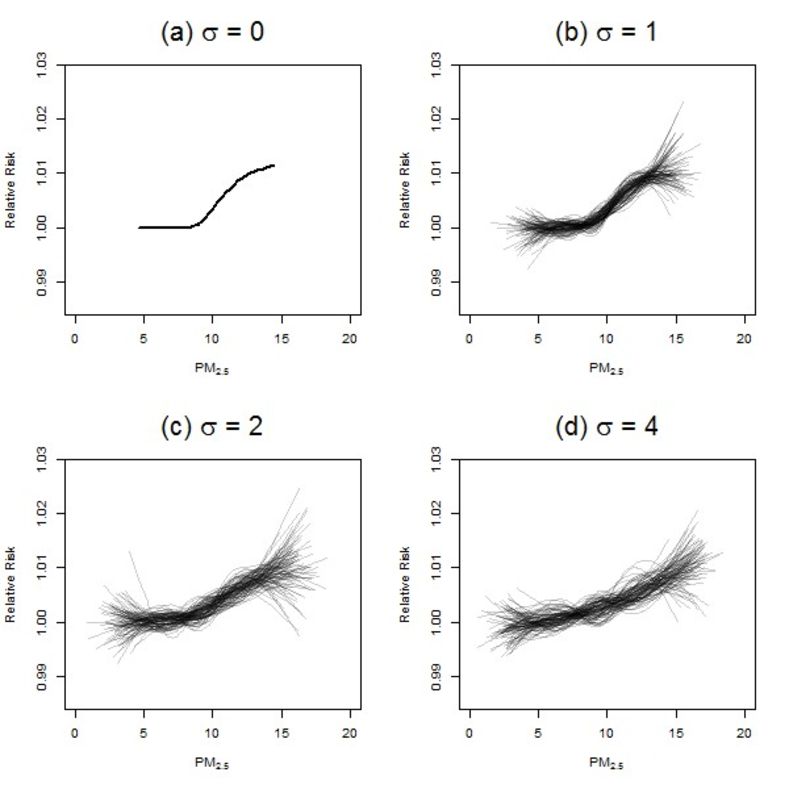

Supplement: S1 Fig — (TIF) [file pone.0264833.s001.tif]

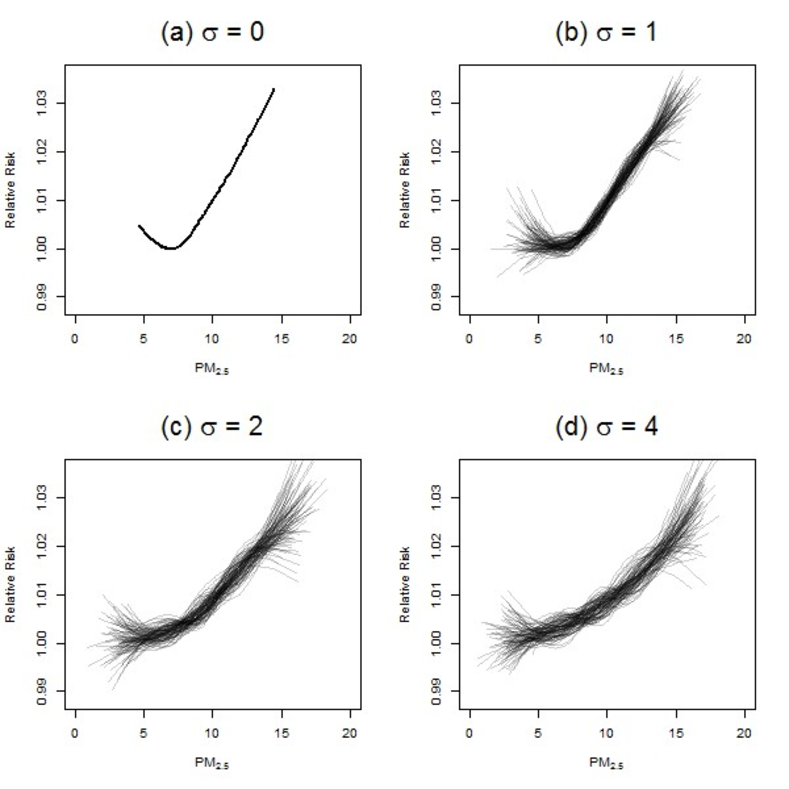

Supplement: S2 Fig — (TIF) [file pone.0264833.s002.tif]

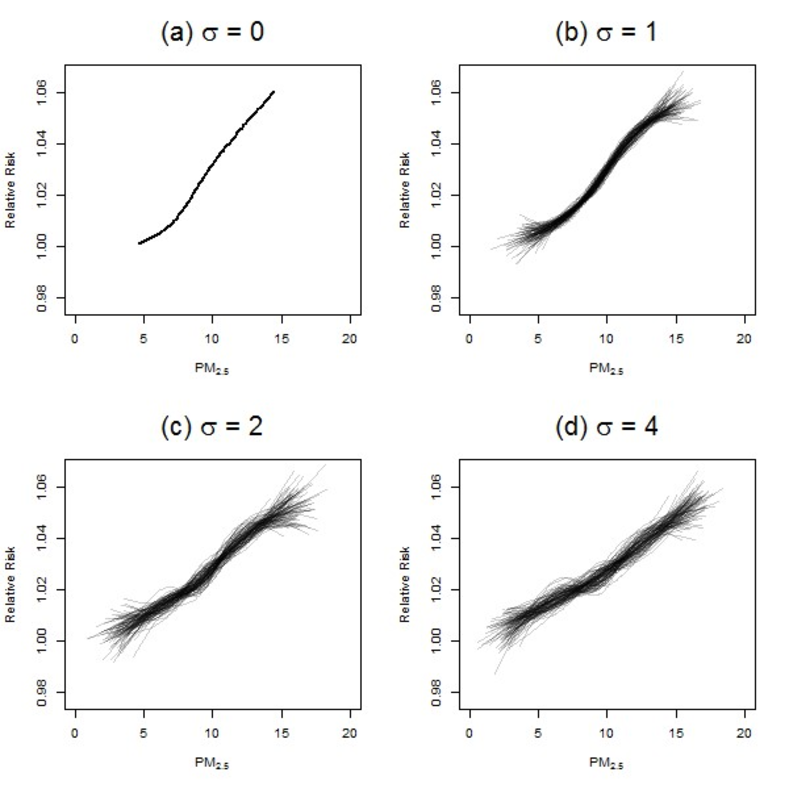

Supplement: S3 Fig — (TIF) [file pone.0264833.s003.tif]

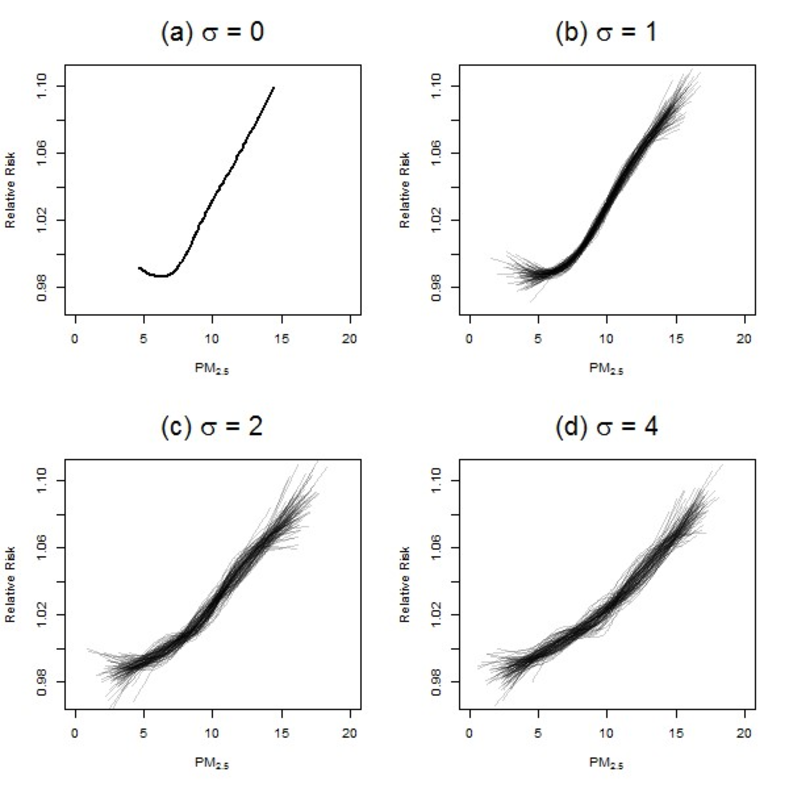

Supplement: S4 Fig — (TIF) [file pone.0264833.s004.tif]

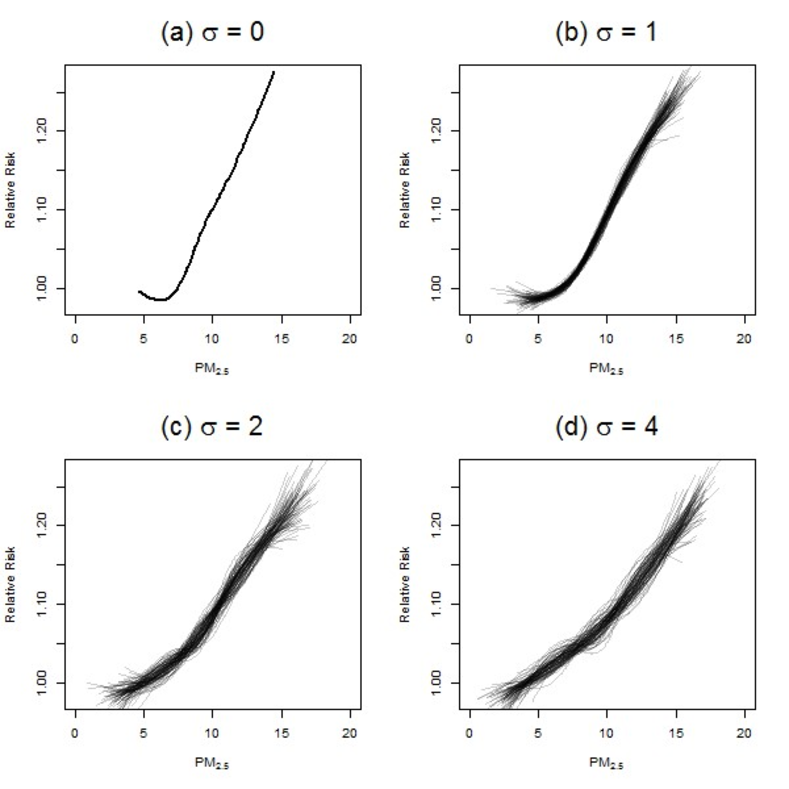

Supplement: S5 Fig — (TIF) [file pone.0264833.s005.tif]

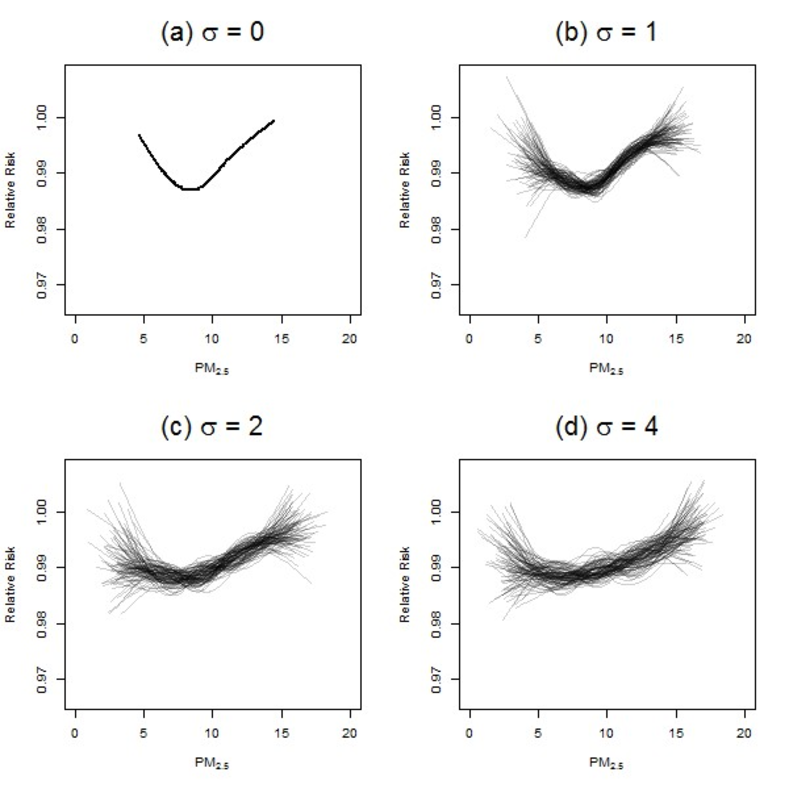

Supplement: S6 Fig — (TIF) [file pone.0264833.s006.tif]

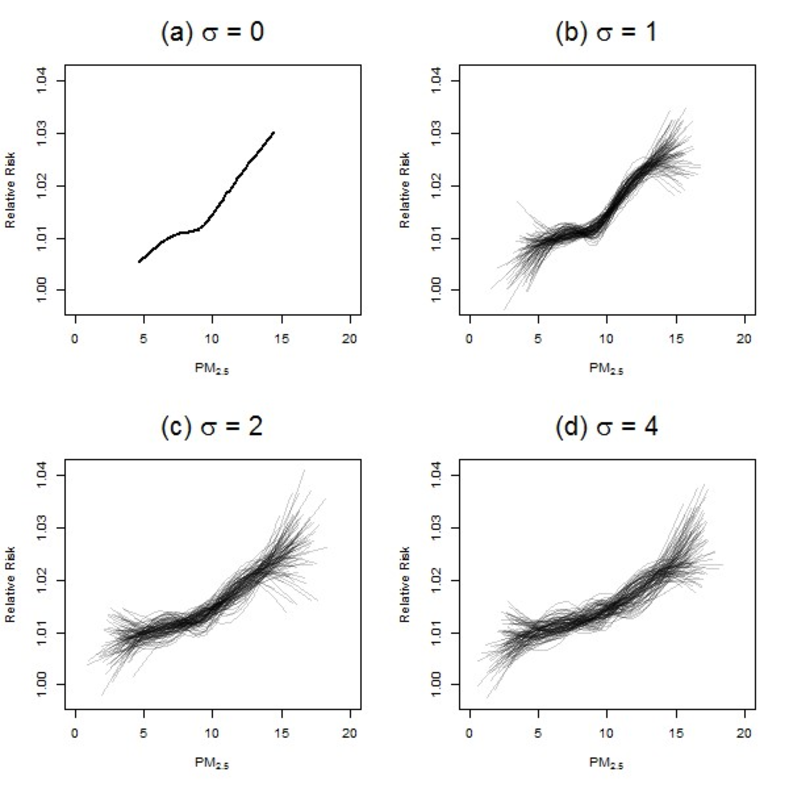

Supplement: S7 Fig — (TIF) [file pone.0264833.s007.tif]

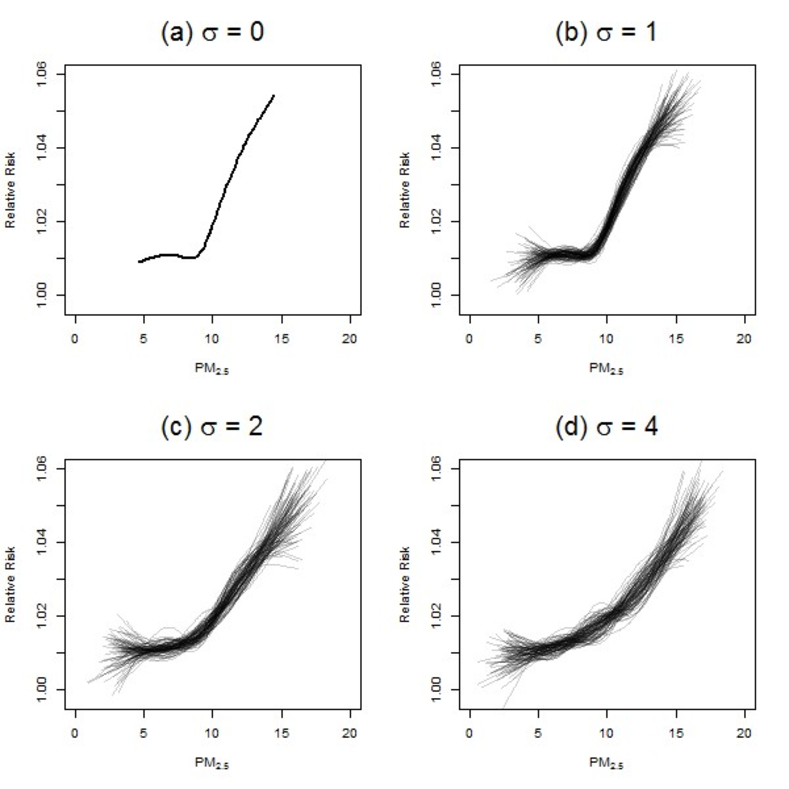

Supplement: S8 Fig — (TIF) [file pone.0264833.s008.tif]

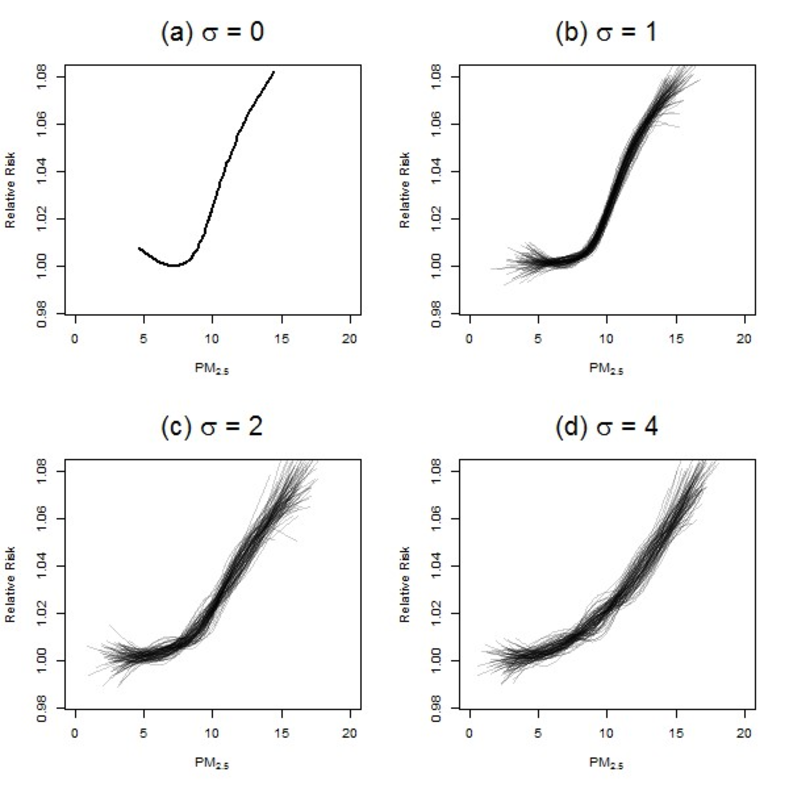

Supplement: S9 Fig — (TIF) [file pone.0264833.s009.tif]

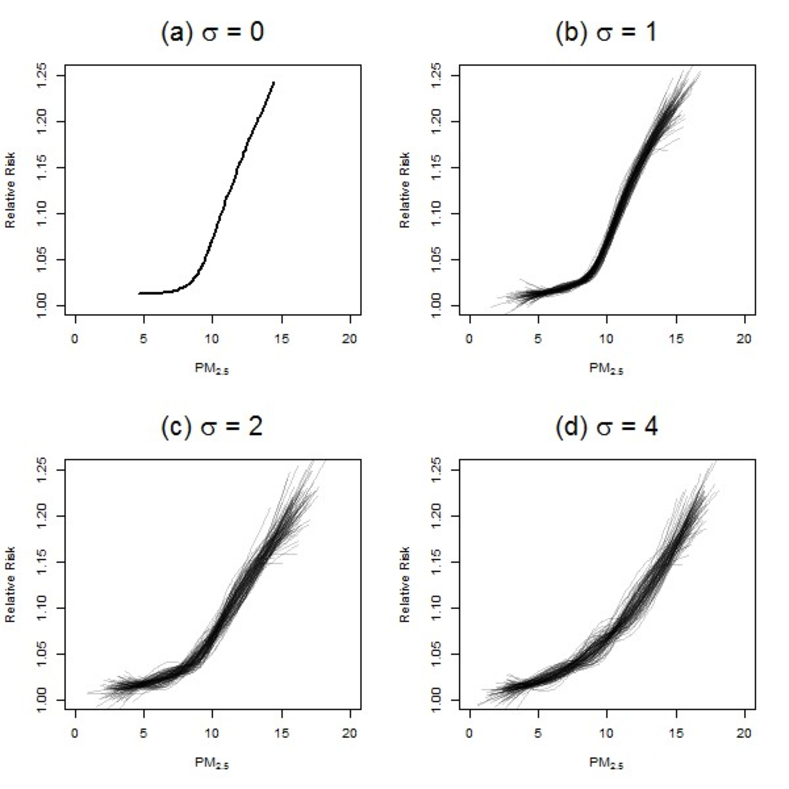

Supplement: S10 Fig — (TIF) [file pone.0264833.s010.tif]

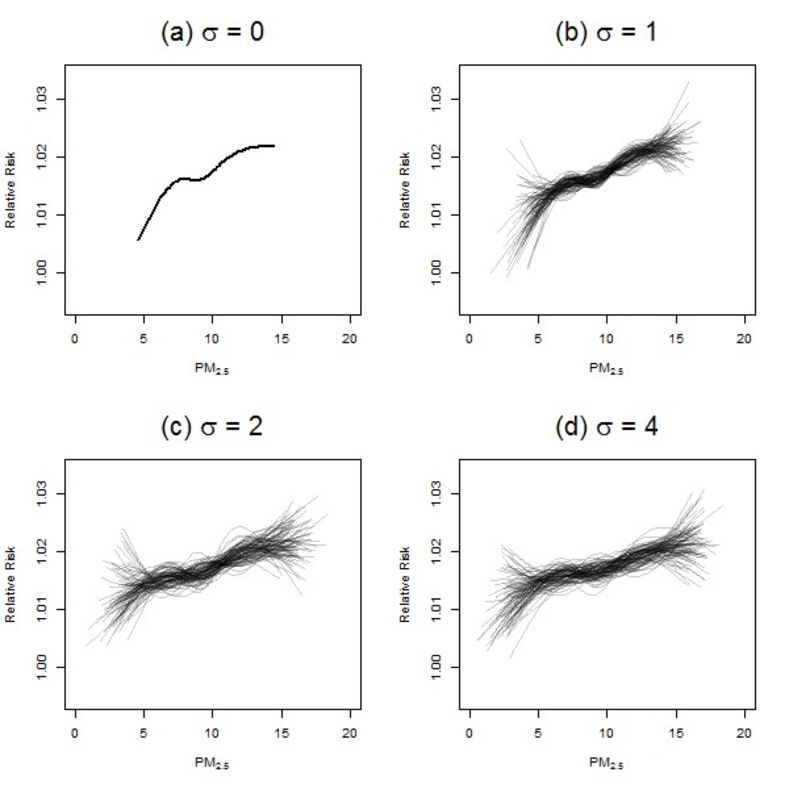

Supplement: S11 Fig — (TIF) [file pone.0264833.s011.tif]

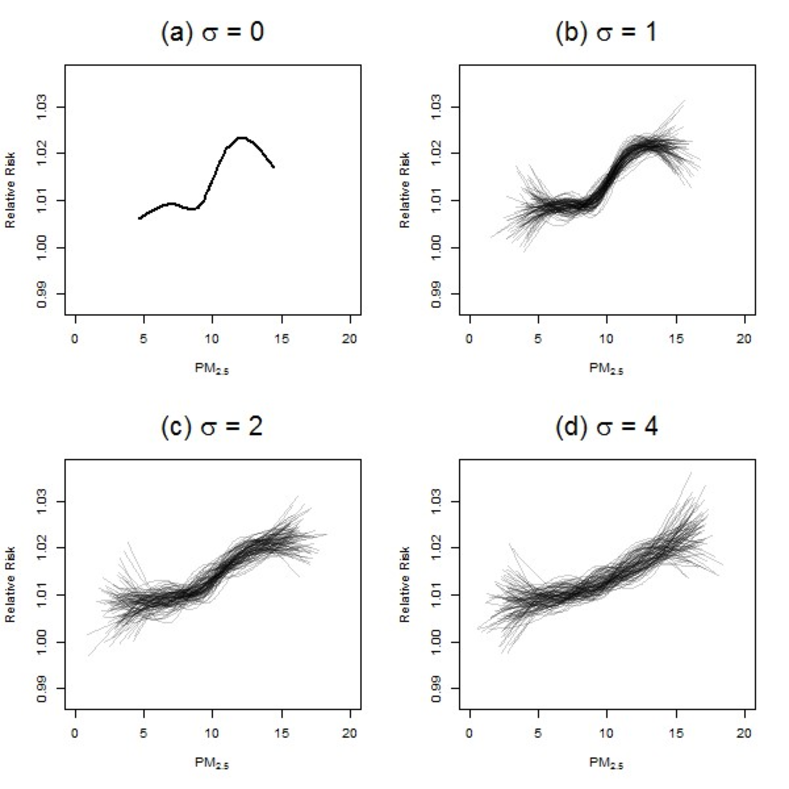

Supplement: S12 Fig — (TIF) [file pone.0264833.s012.tif]

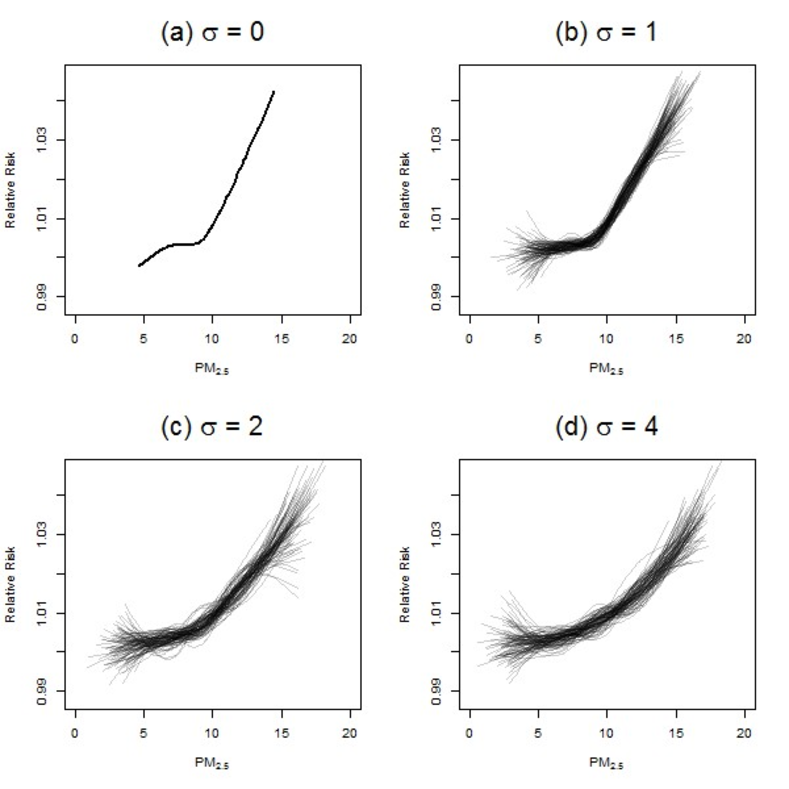

Supplement: S13 Fig — (TIF) [file pone.0264833.s013.tif]

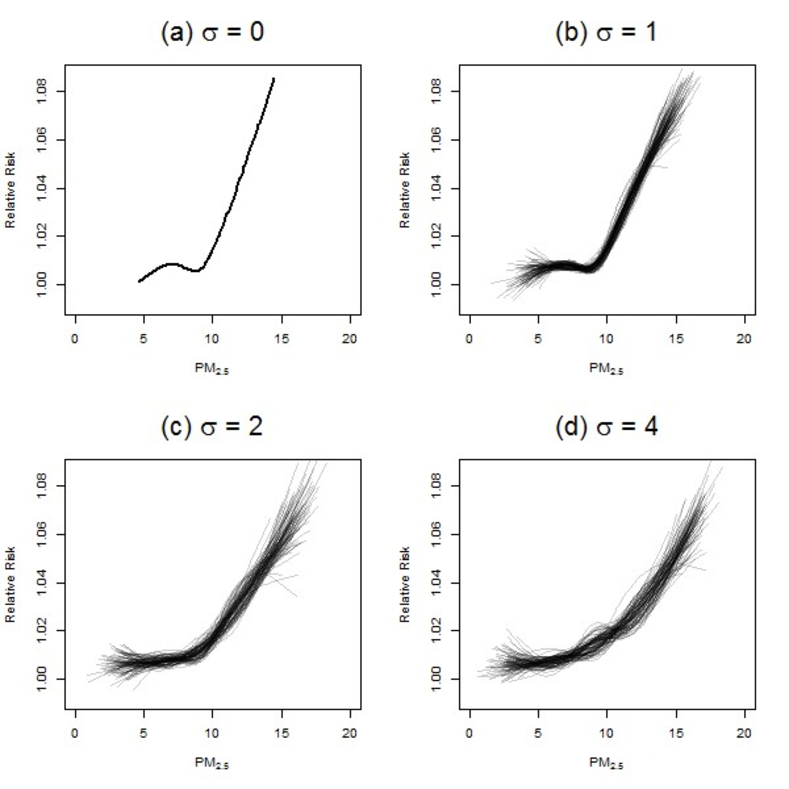

Supplement: S14 Fig — (TIF) [file pone.0264833.s014.tif]

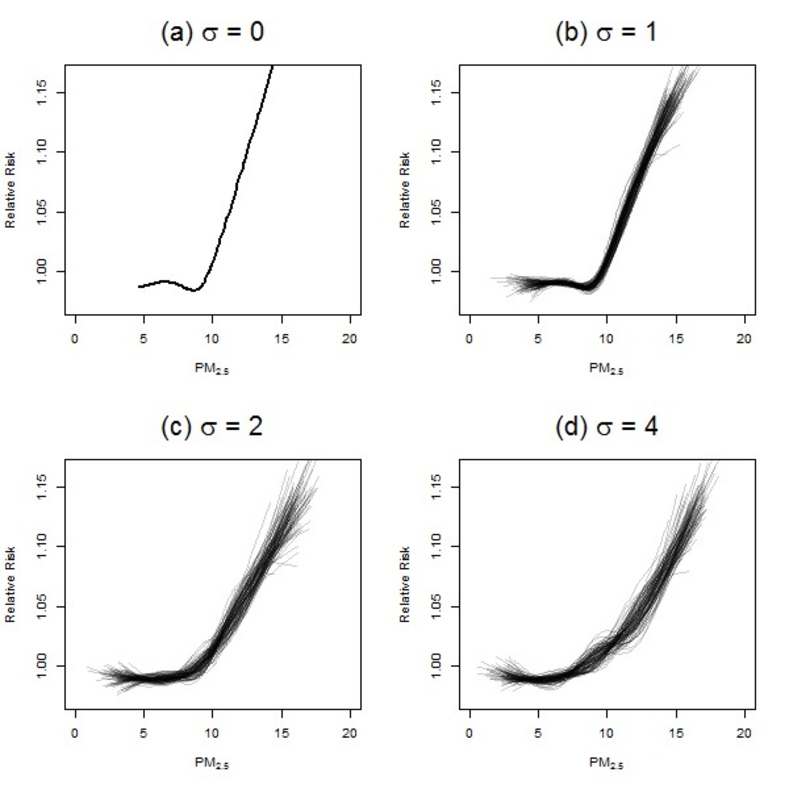

Supplement: S15 Fig — (TIF) [file pone.0264833.s015.tif]
