# Supplementary material for: A simulation-based assessment of the ability to detect thresholds in chronic risk concentration-response functions in the presence of exposure measurement error
Source: PLoS One. 2022 Mar 11;17(3):e0264833. doi: 10.1371/journal.pone.0264833 (PMC8916630; doi:10.1371/journal.pone.0264833)
Supplement: S1 Table — (PDF) [file pone.0264833.s016.pdf]

	Threshold = 7	Threshold = 8.5	Threshold = 9.5
HR = 1.0025			
σ = 1	56	90	2
σ = 2	47	67	13
σ = 4	33	57	10
HR = 1.005			
σ = 1	99	83	74
σ = 2	85	59	51
σ = 4	71	52	51
HR = 1.01			
σ = 1	75	100	100
σ = 2	50	97	87
σ = 4	43	88	81
HR = 1.02			
σ = 1	99	100	100
σ = 2	95	100	100
σ = 4	83	99	99
HR = 1.05			
σ = 1	100	100	100
σ = 2	100	100	100
σ = 4	97	100	100

Note: Each numeric entry is the number of times out of 100 tests that a Cox PH model that assumed the “true” C-R threshold fit the data better than a Cox PH model that assumed no C-R threshold.
