# Supplementary material for: A simulation-based assessment of the ability to detect thresholds in chronic risk concentration-response functions in the presence of exposure measurement error
Source: PLoS One. 2022 Mar 11;17(3):e0264833. doi: 10.1371/journal.pone.0264833 (PMC8916630; doi:10.1371/journal.pone.0264833)
Supplement: S2 Table — (PDF) [file pone.0264833.s017.pdf]

	Threshold = 7	Threshold = 8.5	Threshold = 9.5
HR = 1.0025			
$\sigma = 1$	0	0	0
$\sigma = 2$	0	0	0
$\sigma = 4$	0	0	0
HR = 1.005			
$\sigma = 1$	19	0	1
$\sigma = 2$	9	3	0
$\sigma = 4$	5	0	0
HR = 1.01			
$\sigma = 1$	1	99	71
$\sigma = 2$	3	74	31
$\sigma = 4$	3	39	19
HR = 1.02			
$\sigma = 1$	96	100	100
$\sigma = 2$	67	100	98
$\sigma = 4$	44	86	91
HR = 1.05			
$\sigma = 1$	100	100	100
$\sigma = 2$	95	100	100
$\sigma = 4$	81	100	100

Note: Each numeric entry is the number of times out of 100 tests that a Cox PH model that assumed the “true” C-R threshold fit the data better than a Cox PH model that assumed no C-R threshold.
